# Can metagenomic next-generation sequencing identify the pathogens responsible for culture-negative prosthetic joint infection?

**DOI:** 10.1186/s12879-020-04955-2

**Published:** 2020-03-30

**Authors:** Chaoxin Wang, Zida Huang, Wenbo Li, Xinyu Fang, Wenming Zhang

**Affiliations:** Department of Orthopedic Surgery, The First Affiliated Hospital of Fujian Medical University, No. 20 Chazhong Road, Fuzhou, 350005 China

**Keywords:** Metagenomic next-generation sequencing, Culture-negative, Prosthetic joint infection, Antibiotic

## Abstract

**Background:**

The aims of this study were to (1) evaluate the efficacy and safety of targeted antibiotics for the treatment of culture-negative prosthetic joint infection based on metagenomic next-generation sequencing results and (2) verify the accuracy and reliability of metagenomic next-generation sequencing for identifying pathogens related to culture-negative prosthetic joint infection.

**Methods:**

Ninety-seven consecutive PJI patients, including 27 patients with culture-negative prosthetic joint infection, were treated surgically at our center. Thirteen of the 27 culture-negative prosthetic joint infection patients, who were admitted before June 2017 and treated with empirical antibiotics, comprised the empirical antibiotic group (EA group), and the other 14 patients, who were admitted after June 2017 and treated with targeted antibiotics according to their metagenomic next-generation sequencing results, were classified as the targeted antibiotic group (TA group). The short-term infection control rate, incidence of antibiotic-related complications and costs were compared between the two groups.

**Results:**

Two of the patients in the EA group experienced debridement and prolonged antimicrobial therapy due to wound infection after the initial revision surgery. No recurrent infections were observed in the TA group; however, no significant difference in the infection control rate was found between the two groups (83.33% vs 100%, *P* = 0.217). More cases of antibiotic-related complications were recorded in the EA group (6 cases) than in the TA group (1 case), but the difference was not statistically significant (*P* = 0.0697). The cost of antibiotics obtained for the EA group was 20,168.37 Yuan (3236.38–45,297.16), which was higher than that found for the TA group (10,164.16 Yuan, 2959.54–16,661.04, *P* = 0.04).

**Conclusions:**

Targeted antibiotic treatment for culture-negative prosthetic joint infection based on metagenomic next-generation sequencing results is associated with a favorable outcome, and metagenomic next-generation sequencing is a reliable tool for identifying pathogens related to culture-negative prosthetic joint infection.

## Background

Due to pathogen encapsulation within biofilms [1], low microbial concentrations [2], the prior use of antibiotics [3], and the difficulty associated with the culturing of some pathogens [4], culture-negative prosthetic joint infection (CN-PJI) accounts for approximately 7–12% of all PJI cases [5]. The current guidelines and the consensus opinion recommend the use of empirical therapy with vancomycin and broad-spectrum beta-lactams (piperacillin/tazobactam or third-generation cephalosporins) for the treatment of CN-PJI [6–8]. However, long-term therapy with broad-spectrum antibiotics causes systemic toxicity in patients [9] and is not effective against fungi and some rare pathogens [8], which can potentially lead to treatment failure. In recent years, many studies have identified pathogens in the joint fluid or sonication fluid of PJI patients and verified these by metagenomic next-generation sequencing (mNGS). The results revealed that mNGS can detect new potential pathogens in 16–44% of CN-PJI patients and 4–67% of culture-positive (CP)-PJI patients [10–14] and might be a useful adjunct in the selection of antibiotic protocols. Previous studies have mainly focused on the diagnostic value of mNGS; therefore, the efficacy and safety of antibiotic protocols based on mNGS results remain unknown.

In contrast, the high sensitivity of mNGS poses a problem, particularly due to the large number of microorganisms obtained in almost all mNGS results. Although each study established various thresholds for the abundance or number of reads in attempts to distinguish between pathogens and background microorganisms [10, 15], no good method has been established for verifying whether the microorganisms detected by mNGS were responsible for the PJI. However, the accuracy of the pathogen diagnosis can be indirectly verified if the choice of antibiotic treatment selected according to mNGS results is successful.

Therefore, this study compared the clinical outcomes of a group of CN-PJI patients administered targeted antibiotics based on mNGS results and a group of CN-PJI patients receiving empirical antibiotics. The aims of this study were to (1) evaluate the efficacy and safety of mNGS-based antibiotic treatment for CN-PJI and (2) verify the accuracy and reliability of mNGS for identifying pathogens related to CN-PJI.

## Methods

### Study population

Ninety-seven patients with a first episode of PJI, including 27 patients with CN-PJI, were admitted and treated surgically in our center from January 2016 to December 2018. These patients included 13 males and 14 females, and the infections were located in 17 hips and 10 knees. In addition, the average patient age was 65.7 (65.7 ± 14.63) years. mNGS was not performed prior to 2017; thus, 13 patients with CN-PJI were treated with empirical antibiotics and comprised as the empirical antibiotic group (EA group), and the other 14 patients with CN-PJI, who were admitted after June 2017, were treated with targeted antibiotics according to mNGS results and comprised the targeted antibiotic group (TA group). The study was approved by the institutional review board (ethical approval number: 2018–026).

### Definition of CN-PJI

The diagnosis of PJI was based on the revised diagnostic criteria delineated by the Musculoskeletal Infection Society [16]. The characteristics and laboratory results are provided in Additional file 1. If the same microorganism was not isolated from any two separate samples of tissue, synovial fluid or sonication fluid from a patient, the patient was classified as CN.

### Surgical therapy

Patients classified as Tsukayama type II and type III were subjected to irrigation and debridement (I&D) [17], whereas two-stage revision surgery was routinely performed on the Tsukayama type I and type IV patients, and the patients with substantial medical comorbidities and no sinus at the wound were subjected to one-stage revision in accordance with the patient’s condition.

All procedures were performed by the same group of surgeons. (a) During I&D, the surgeons completely removed the necrotic tissue, replaced the polyethylene insert, and irrigated the area with a large amount of iodophor and saline. (b) During one-stage revision, the surgeons removed all prosthetic components and necrotic tissue, performed irrigation, and implanted the new prosthesis. During two-stage revision, the surgeons completed stage I debridement and placed an antibiotic-impregnated cement spacer. If the pathogens were not identified prior to surgery, 2–4 g of vancomycin and 0.5 g of gentamicin were added per 40 g of bone cement. If mNGS results were obtained prior to the operation, 2–4 g of targeted antibiotics were added per 40 g of bone cement. Finally, the new prosthesis was implanted after re-debridement during two-stage surgery.

### Microbiology culture

For culture, 0.1-ml aliquots of joint fluid and sonication fluid were incubated on blood agar at 35–37 °C in 5–7% CO_2_ under aerobic and anaerobic conditions for 7 and 14 days, respectively. The residual samples were inoculated into a BACTEC Peds Plus/F bottle, incubated in a BD automatic incubator (Becton-Dickinson, Germany) under aerobic and anaerobic conditions for 5 and 14 days, respectively, and subcultured on blood agar if a positive result was obtained. The tissue was homogenized with broth and inoculated under the above-described protocol. All bacterial identification was performed using a Vitek 2 system (BioMerieux Vitek, Inc., USA).

### Sequence and analysis

The protocol has been reported previously [14]. Briefly, (1) we utilized ceramic beads to break the cell wall extracted total DNA from synovial fluid, sonication fluid or homogenized tissue using a TIANamp Micro DNA Kit (DP316, Tiangen Biotech). (2) We sonicated the DNA to generate 200–300-bp fragments, and DNA nanospheres were prepared by PCR amplification and circularization reactions and sequenced on a BGISEQ-500 platform (BGI-Wuhan, China) with a 50-bp single-end (SE50) format. Then we proceeded samples in batches with the negative control (double distilled H2O), and it will be reprocessed from nucleic acid extraction if contamination was highly suspected. Contamination conditions commonly include detection of pathogens which were not common reagent-related from negative control samples, or simultaneous detection of same pathogens with high number of reads from most samples in a batch. (3) We analyzed the original sequencing data using a bioinformatic pipeline developed by BGI. We removed the human host sequences and compared the obtained sequences with the in-house Microbial Genome database built by BGI Company to obtain a list of bacterial and fungal species. (4) We then distinguished the potential pathogens with background signals and microorganisms based on their relative abundances and numbers of reads. A detailed workflow is provided in the Additional file 2.

### Antibiotic therapy

All cases were treated for approximately 12 weeks regardless of the type of surgical procedure. The duration of intravenous antibiotic treatment was determined based on the clinical symptoms, signs and serological indicators of the patient. The treatment was adjusted to oral antibiotics until the end of the antibiotic therapy if the patient exhibited no symptoms or signs of infection recurrence, decreasing C-reactive protein (CRP) levels, a declining erythrocyte sedimentation rate (ESR) and had been subjected to intravenous antibiotic treatment for more than 2 weeks.

For the EA group, the intravenous antibiotic protocol was vancomycin combined with a third-generation cephalosporin or quinolone antibiotic. The oral antibiotic protocol was rifampicin combined with quinolone antibiotics.

For the TA group, the intravenous antibiotic and oral antibiotic protocols were the same. Empirical antibiotic treatment was administered until the mNGS results were obtained, and targeted antibiotics were then selected according to the mNGS results and opinions of the multidisciplinary team.

### Prognosis evaluation

All patients were regularly monitored for routine blood parameters, liver function, and renal function during antibiotic treatment. The patients were followed at postoperative intervals of 1 month, 3 months, and 1 year, and their symptoms, signs, and serological indicators were recorded to determine the treatment outcome.

Infection control was assessed using the Delphi standard [18]. The infection control rate was defined as the proportion of patients who exhibited infection control in the group. The definition of an antibiotic-related complication was based on the 2017 Common Terminology Criteria for Adverse Events (CTCAE).

### Statistical analysis

Student’s t-tests were employed if the measurement data exhibited a normal distribution, whereas Mann-Whitney U-tests were utilized if the data did not follow a normal distribution. The enumeration data were analyzed by a chi-square test or Fisher’s exact test. The statistical analyses were performed using SPSS v25.0 (SPSS Inc., USA), and *P* < 0.05 was considered to indicate statistical significance.

## Results

### Included population

One patient in the EA group was lost to follow-up, and a total of 12 patients were included in this group. The average follow-up time for this group was 39 (34–44) months. One patient in the TA group was lost to follow-up, and one patient in this group was not treated according to their mNGS results due to suspected contamination during sample transportation. A total of 12 cases were ultimately included in the TA group, and the average follow-up time for this group was 18.5 (12–32) months. The demographic characteristics, with the exception of the follow-up time, showed no significant differences between the two groups (Table 1).

**Table 1:** Demographic characteristics of the EA and TA groups

Variable	EA group (*n* = 12)	TA group (*n* = 12)	*P* value
Sex, male	3	8	0.1^*^
Age (years)	60.67 ± 14.705	70.67 ± 13.296	0.095^***^
Joint, hip	9	6	0.4^**^
BMI (kg/m^2^)	24.46 ± 4.217	25.16 ± 4.30	0.71^***^
CCI	3	3.83	0.68^***^
Sinus	15	7	1^**^
CRP	38.3 ± 63.883	63.49 ± 34.163	0.891^***^
ESR	68.92 ± 38.901	63.49 ± 42.324	0.747^***^
Aspirate WBCs (/cmm)	5834 (2219–14,818)	9441.5 (1100–31,810)	0.091^****^
Aspirate neutrophils (%)	83.30 ± 11.531	81.01 ± 15.08	0.681^***^
Tsukayama type			
II	5	3	
IV	7	9	0.667^**^
Follow-up (month)	39 (34–44)	18.5 (12–32)	0.001^****^

*BMI* body mass index, *ASA* American Society of Anesthesiologists, *CCI* Charlson Comorbidity Index, *CRP* C-reactive protein, *ESR* erythrocyte sedimentation rate, *WBC* white blood cell

^*^Chi-square test; ^**^Fisher’s exact test; ^***^Student’s t-test; ^****^Mann-Whitney U-test

### mNGS results

Nine joint fluid samples, two sonication fluid samples, and one tissue sample from the patients in the TA group were subjected to mNGS. The average total read number of each sample was 26,381,264.5 (12,930,006-45,006,663), and the average pathogen read number was 148 (3–2240). Twelve cases were identified as infections caused by a single pathogen. The read number and genome coverage of each sample are provided in the supplementary materials.

### Antibiotic management

The antibiotic-related costs and duration of intravenous antibiotic treatment in the TA group were significantly higher than those in the EA group (Table 2). The overall incidence of antibiotic complications in the EA group was 50% (6/12), and these complications included liver dysfunction in three patients and renal dysfunction, leukopenia, and drug eruption in the remaining patients who experienced complications. Only one case of renal dysfunction was observed in the EA group, and the overall antibiotic complication rate in this group was 8.33% (1/12). More antibiotic-related complications were detected in the EA group than in the TA group, but the difference was not statistically significant (8.33% vs 50%, *P* = 0.069).

**Table 2:** Antibiotic management protocols for the EA and TA groups

Variable	EA group (*n* = 12)	TA group (*n* = 12)	*P* value
Intravenous antibiotic duration (d)	22.92 (13–35)	16.58 (13–24)	0.891^*^
Antibiotic costs (Yuan)	20,168.37 (3236.38–45,297.16)	10,164.16 (2959.54–16,661.04)	0.043^*^
Antibiotic-related complications	6	1	0.091^**^
Liver dysfunction	3	0	0.59^**^
Renal dysfunction	1	1	0.478^**^
Leukopenia	1	0	1^**^
Drug eruption	1	0	1^**^

^*^Mann-Whitney U test;^**^Chi-square test

### Outcomes

The overall infection control rate in the EA group was 83.33% (10/12). Two cases of persistent incision drainage occurred on the 9th and 10th days after the initial surgery. I&D was performed immediately, and no recurrence was subsequently observed (Table 3). In the TA group, no recurrence was observed during the study period, and the overall infection control rate was 100% (12/12) (Table 4). The infection control rate of the TA group was higher than that of the EA group, but the difference in the infection control rate between the two groups was not significant (100% vs 75%, *P* = 0.093).

**Table 3:** Treatment factors and results of the EA group

Case	Tsukayama type	Surgical protocol	Antibiotic regimen		Reason for culture-negative	Follow-up (month)	Subsequent intervention	Antibiotic costs (Yuan)
			Intravenous	Oral				
EA-1	IV	Two-stage revision	Vancomycin Ceftazidime	Levofloxacin Rifampicin	Unknown	34	–	8587.82
EA-2	IV	Two-stage revision	Vancomycin Meropenem	Levofloxacin Rifampicin	Unknown	34	I&D	39,572.64
EA-3	IV	Two-stage revision	Vancomycin Ceftazidime	Levofloxacin Rifampicin	Unknown	38	–	16,837.93
EA-4	IV	Two-stage revision	Vancomycin Ceftazidime	Levofloxacin Rifampicin	Unknown	37	–	6697.8
EA-5	II	I&D	Vancomycin Levofloxacin	Levofloxacin Rifampicin	Prior use of antibiotics	43	I&D	23,050.38
EA-6	II	I&D	Vancomycin Meropenem	Levofloxacin Rifampicin	Prior use of antibiotics	40	Suppressive antibiotic therapy	30,235.4
EA-7	IV	One-stage revision	Vancomycin Ceftazidime	Levofloxacin Rifampicin	Prior use of antibiotics	37	–	3236.38
EA-8	II	I&D	Vancomycin Ceftazidime	Levofloxacin Rifampicin	Unknown	42	–	9051.3
EA-9	II	I&D	Vancomycin Meropenem	Levofloxacin Rifampicin	Prior use of antibiotics	41	–	45,297.16
EA-10	II	I&D	Vancomycin Ceftazidime	Levofloxacin Rifampicin	Unknown	44	–	6583.4
EA-11	IV	Two-stage revision	Vancomycin Moxifloxacin	Levofloxacin Rifampicin	Unknown	38	–	13,756.7
EA-12	IV	Two-stage revision	Vancomycin Ceftazidime	Levofloxacin Rifampicin	Prior use of antibiotics	41	I&D	39,113.54

**Table 4:** Treatment factors and results of the TA groups

Case	Tsukayama type	Surgical protocol	mNGS results	Intravenous antibiotic regimen		Oral antibiotic regimen	Reason for culture-negative result	Follow-up (month)	Subsequent intervention	Antibiotic costs (Yuan)
				Initial	Targeted					
TA-1	IV	Two-stage revision	*Staphylococcus aureus*	Vancomycin Meropenem	Vancomycin	Levofloxacin Rifampicin	Prior use of antibiotics	24	–	11,146.6
TA-2	IV	Two-stage revision	Parvimonas micra	Vancomycin Imipenem	Amoxicillin Clavulanate	Amoxicillin/Clavulanate	Fastidious bacteria	23	–	10,421
TA-3	IV	Two-stage revision	Corynebacterium	Vancomycin Meropenem	Vancomycin	Levofloxacin Rifampicin	Prior use of antibiotics	32	–	16,661.04
TA-4	IV	Two-stage revision	*Staphylococcus epidermidis*	Vancomycin Ceftazidime	Vancomycin	Linezolid	Prior use of antibiotics	30	–	10,312.27
TA-5	II	I&D	Mycoplasma hominis	Vancomycin Meropenem	Levofloxacin	Doxycycline Levofloxacin	Fastidious bacteria	18	–	8391.27
TA-6	II	I&D	Mycoplasma hominis	Vancomycin Meropenem	Levofloxacin	Doxycycline Levofloxacin	Fastidious bacteria	27	–	8391.27
TA-7	II	One-stage revision	*Streptococcus agalactiae*	Vancomycin Ceftazidime	Ceftriaxone	Levofloxacin	Unknown	18	–	11,946.99
TA-8	IV	I&D	Staphylococcus epidermidis	Vancomycin Levofloxacin	Vancomycin	Moxifloxacin Rifampicin	Unknown	27	–	11,016.02
TA-9	IV	I&D	Mycoplasma hominis	Vancomycin Ceftazidime	Levofloxacin	Levofloxacin Doxycycline	Unknown	27	–	2959.54
TA-10	IV	I&D	Staphylococcus epidermidis	Vancomycin Tazocin	Vancomycin	Linezolid	Unknown	19	–	9298.5
TA-11	IV	Two-stage revision	Finegoldia magna	Vancomycin Meropenem	Vancomycin Tazocin	Amoxicillin Clavulanate	Fastidious bacteria	17	–	15,499.49
TA-12	IV	Two-stage revision	*Candida tropicalis*	Vancomycin	Fluconazole	Fluconazole	Fastidious bacteria	12	–	5925.74

## Discussion

Previous studies have suggested that negative culture results might reduce the infection control rate of PJI and increase the complication rate associated with broad-spectrum antibiotics [19]. This study compared the efficacy and safety of targeted antibiotic therapy with empirical antibiotic therapy in patients with CN-PJI. The results showed that antibiotic-related complications, duration of intravenous antibiotic treatment and antibiotic costs obtained for the TA group were lower than those found for the EA group. These results indirectly demonstrated the accuracy of mNGS in the diagnosis of patients associated with CN-PJI and that mNGS can be used to aid clinical decisions. Our center has used a variety of methods, such as prolonged culture time, automatic blood culture bottles, and sonication, to optimize microbial cultivation since 2016. Therefore, we included cases from January 2016 to ensure the conformity of the two groups. Twenty-seven cases of CN-PJI cases (27.84%) were recorded among the 97 consecutive PJI cases during the study period, and this rate is similar to that reported in the literature [5, 20]. This result reflects the actual situation at the PJI referral center to some extent.

A total of 24 CN-PJI cases were included in the study. The overall infection control rate in the EA group was 83.33% (10/12), which is similar to that obtained in previous studies [21–23]. This rate might be due to the fact that CN-PJI is often caused by rare pathogens, and empirical antibiotic treatment does not effectively treat these pathogens [8]. However, 12 cases in the TA group exhibited no recurrence until the last follow-up. The pathogens detected by mNGS in the samples from the 12 CN-PJI patients included many rare pathogens and fastidious bacteria, such as *Mycoplasma hominis* (3 cases), *Finegoldia magna* and *Parvimonas micra*. Therapy with sensitive antibiotics can easily control infection after surgery as well as avoid the systemic toxicity and expensive costs associated with long-term therapy using broad-spectrum antibiotics. Interestingly, *Candida albicans* was also successfully detected by mNGS. Fungal PJI is rare and refractory [24], and the empirical antibiotic protocols for CN-PJI are not applicable to fungal infections. In the absence of the information provided by mNGS would, patients with PJI caused by *Candida albicans* are likely to exhibit infection recurrence leading to surgical failure. mNGS also successfully detected three cases of *Staphylococcus epidermidis* and one case of *Staphylococcus aureus*. Because half of the isolates were methicillin-resistant *Staphylococcus aureus* and the drug susceptibility was unknown, the protocol for EA therapy used in this study consisted of vancomycin and broad-spectrum beta-lactams. This protocol is effective for almost all drug-resistant bacteria, which might explain the lack of significantly different infection control rates between the two groups.

Leukopenia, liver dysfunction, renal dysfunction, and drug eruption are the most common complications of PJI EA therapy [9], and thus, this study regularly monitored for these complications during antibiotic therapy. The results showed that the patients in the TA group experienced a lower incidence of antibiotic-related complications than those in the EA group, although no significant difference in the complication rate was found between the two groups, which might be due to the small sample size. The duration of intravenous antibiotic treatment in the TA group was significantly longer than that in the EA group because the sensitive antibiotics were more effective in targeting the pathogens than the broad-spectrum antibiotics. Hospitals in China have banned intravenous infusion for outpatients, and the treatment of CN-PJI patients based on mNGS will shorten the duration of intravenous antibiotic therapy, reduce the hospitalization period, and improve patient compliance. The cost of antibiotics obtained for the TA group was also significantly lower than that found for the EA group because the patients in the TA group were intravenously treated with antibiotics for a significantly longer duration than that those in the EA group and because the cost of broad-spectrum antibiotics is higher than that of sensitive antibiotics. Although mNGS was previously expensive, the actual cost of mNGS in our lab is approximately 2500 Yuan, which makes this technique accessible for clinical use. When treating CN-PJI with broad-spectrum antibiotics in clinical practice, we should closely monitor various antibiotic-related side effects, obtain evidence of pathogens in a timely manner and accurately adjust the antibiotic protocol to reduce both the duration of intravenous antibiotic treatment and the incidence of antibiotic-related complications.

mNGS has shown great promise in the diagnosis of PJI and can be used as an effective tool for the identification of pathogens [13, 14]. Some existing studies have indicated that mNGS is particularly useful in cases with strong evidence of infection but negative results from culture or other tests [25]. However, previous studies were unable to directly confirm whether the positive mNGS results obtained for CN-PJI patients were pathogens. Although each study set thresholds for distinguishing between pathogens and background microorganisms, the methods varied, and no standardized quality control standards have been established [10, 15]. In this study, the 12 patients in the TA group achieved better infection control than the patients in the EA group based on the mNGS results. The accuracy of the mNGS diagnosis of the pathogens related to CN-PJI was indirectly verified, and this method can be used to identify pathogens and administer causal treatment early. Hence, further research should focus on the curative effects and costs associated with the application of mNGS for the selection of an appropriate targeted antibiotic.

Tis study has some limitations. First, information bias is inevitable due to the retrospective nature of the investigation. Second, the sample size (only 12 patients in the EA group and 12 patients in the TA group) is very small due to the low incidence of CN-PJI, and there was a certain degree of selection bias. Third, the short-term follow-up of the cases might result in the omission of a small number of cases of recurring infection. Randomized clinical trials might be needed in the future to confirm the clinical utility of the technique.

## Conclusions

In summary, our results indicate that mNGS is reliable for identifying pathogens in patients with CN-PJI and that targeted antibiotic treatment based on mNGS results yields a favorable outcome in a short period of time.

## Supplementary information

**Additional file 1.** Demographic characteristics of the patients in the EA and TA groups.

**Additional file 2.** Metagenomic next-generation sequencing procedures.

## Data Availability

The datasets generated during the current study are available in the CNSA (https://db.cngb.org/cnsa/) of CNGBdb with accession number CNP0000915.

## Abbreviations

CN-PJI: Culture-negative prosthetic joint infection
CP-PJI: Culture-positive prosthetic joint infection
mNGS: metagenomic next-generation sequencing
EA: Empirical antibiotic
TA: Targeted antibiotic
I&D: Irrigation and debridement
CRP: C-reactive protein
CTCAE: Common Terminology Criteria for Adverse Events
