# Supplementary material for: Can metagenomic next-generation sequencing identify the pathogens responsible for culture-negative prosthetic joint infection?
Source: BMC Infect Dis. 2020 Mar 30;20:253. doi: 10.1186/s12879-020-04955-2 (PMC7106575; doi:10.1186/s12879-020-04955-2)
Supplement: Supplementary file 2 — Additional file 2. Metagenomic next-generation sequencing procedures. [file 12879_2020_4955_MOESM2_ESM.docx]

**Metagenomic next-generation sequencing procedures.**

1. Ceramic beads were utilized to break the cell wall. Total genomic DNA was extracted by using TIANamp Micro DNA Kit (DP316, Tiangen Biotech).
2. The extracted DNA was then sonicated to generate 200-300 bp fragments by Covaris S220 (Covaris, Inc., Wobrun, Massachusetts, USA).
3. DNA libraries were constructed according to the standard protocol of the BGISEQ-500 sequencing platform (BGI-Tianjin, Tianjin, China).
4. The quantified libraries were processed for 50bp single-end sequencing on the BGISEQ-500 platform.
5. An aliquot of negative control specimen (whole blood sample taken from healthy donors, proved by isolated mNGS analysis) prepared alongside each batch to monitor contamination. If highlysuspicious contamination occurred, the involved batch will be reanalyzed from initial extraction.

**Microbial Genome Database.**

An in-house database was built by BGI company, which was with no public plans. The complete reference genomes of corresponding species were downloaded from NCBI ftp site (ftp://ftp.ncbi.nih.gov/genomes/). The plasmid sequence and any other non-human-related sequences were removed, keeping only one best genome sequence representing every species based on the assembly results. The alignment index of the reference sequence was built for classifying sequenced reads. The database contains the genomic sequences of 2,700 viruses, 1,494 bacteria, 73 fungi, and 48 parasites that are all related to human diseases.

**Bioinformatics pipeline.**

1. Clean reads of high-quality sequencing data were generated by filtering out the short(45nt and mismatch sites ≤2nt was defined as the mapping quality cut-off.
2. Burrows-Wheeler alignment (BWA, http://bio-bwa.sourceforge.net) was utilized in this pipeline for aligner, match sites >45nt and mismatch sites ≤2nt was defined as the mapping quality cut-off.
3. Human host sequences were eliminated by mapping to the human reference genome (hg19)
4. The remaining sequencing data were aligned to the Microbial Genome Database.
5. SoapCoverage (http://soap.genomics.org.cn/) was utilized to calculate the coverage rate of each species.

**Interpretation of mNGS result.**

The number of raw reads is varied among different samples. In order to reach an equalized comparison, the standardized ratio (SR) is defined as number of total reads/20,000,000. 20,000,000 was setting as a target number of reads when sequencing. All the original number of reads stringently mapped to pathogen in genus-level (SMRNG) or species-level (SMRN) were calculated to standardized number as SDSMRNG and SDSMRN. Coverage rate was defined as SMRN × 50bp /reference genome length.

**Equations**

$$SR=\frac{Number of total reads}{20,000,000}$$

$$SDSRMN=\frac{\mathrm{SMRN}}{\mathrm{SR}}$$

$$SDSRMNG=\frac{\mathrm{SMRNG}}{\mathrm{SR}}$$

$$Coverage rate=\frac{\mathrm{SMRN}\times50}{Reference genome length}$$

**Example**

| Number of total reads | 22341761 |
| --- | --- |
| SR | 1.12 |
| Genus | Staphylococcus |
| Species | Staphylococcus aureus |
| SMRNG | 1730 |
| SMRN | 1502 |
| SDSMRNG | 1549 |
| SDSMRN | 1345 |
| Reference genome length (bp) | 2898306 |
| Coverage rate | 2.59% |

Optimal thresholds were setup as below in order to identify true pathogens.

1. SDSMRNG<3 was considered as insignificant, except for Mycobacterium tuberculosis complex (MTC), while any aligned read of MTC was considered positive.
2. *Burkholderia, Ralstonia, and Delftia* were considered as positive when relative abundance in genus level >=80 %, since they were regarded as most common contamination genus in the lab, and rarely been cultured or validated by specific PCR as pathogen in microbiology lab.
3. The relative abundance in genus level >=15% were determined as the optimal threshold for bacterial identification, while relative abundance in genus level >=30% for fungi, as indicated in the main body.
4. Microbial species whose coverage rate was of the first rank within positive genus and with SDSMRN >=3 was determined as positive species.
